# Genetic structure in the southernmost populations of black-and-gold howler monkeys (*Alouatta caraya*) and its conservation implications

**DOI:** 10.1371/journal.pone.0185867

**Published:** 2017-10-02

**Authors:** Luciana Inés Oklander, Carolina Isabel Miño, Gabriela Fernández, Mariela Caputo, Daniel Corach

**Affiliations:** 1 Instituto de Biología Subtropical (IBS), Nodo Iguazú, Universidad Nacional de Misiones (UNaM) – CONICET and Asociación Civil Centro de Investigaciones del Bosque Atlántico (CeIBA), Puerto Iguazú, Misiones, Argentina; 2 Centro de Bioinvestigaciones – CeBio, Universidad Nacional del Noroeste de la Provincia de Buenos Aires (UNNOBA) – CONICET, Pergamino, Buenos Aires, Argentina; 3 Servicio de Huellas Digitales Genéticas, Facultad de Farmacia y Bioquímica, Universidad de Buenos Aires (UBA) – CONICET, Ciudad Autónoma de Buenos Aires, Argentina; National Cheng Kung University, TAIWAN

## Abstract

Black-and-gold howler monkeys *Alouatta caraya*, are arboreal primates, inhabitants of Neotropical forests, highly susceptible to the yellow fever virus, considered early 'sentinels' of outbreaks, and thus, of major epidemiological importance. Currently, anthropogenic habitat loss and modifications threatens their survival. Habitat modification can prevent, reduce or change dispersal behavior, which, in turn, may influence patterns of gene flow. We explored past and contemporary levels of genetic diversity, elucidated genetic structure and identified its possible drivers, in ten populations (*n* = 138) located in the southernmost distribution range of the species in South America, in Argentina and Paraguay. Overall, genetic variability was moderate (ten microsatellites: 3.16 ± 0.18 alleles per locus, allelic richness of 2.93 ± 0.81, 0.443±0.025 unbiased expected heterozygosity; 22 haplotypes of 491-bp mitochondrial Control Region, haplotypic diversity of 0.930 *±* 0.11, and nucleotide diversity of0.01*±* 0.007). Significant evidence of inbreeding was found in a population that was, later, decimated by yellow fever. Population-based gene flow measures (*F*_*ST*_ = 0.13; *θ*_*ST*_ = 018), hierarchical analysis of molecular variance and Bayesian clustering methods revealed significant genetic structure, grouping individuals into four clusters. Shared haplotypes and lack of mitochondrial differentiation (non-significant *θ*_*ST*_) among some populations seem to support the hypothesis of historical dispersal via riparian forests. Current resistance analyses revealed a significant role of landscape features in modeling contemporary gene flow: continuous forest and riparian forests could promote genetic exchange, whereas disturbed forests or crop/grassland fields may restrict it. Estimates of effective population size allow anticipating that the studied populations will lose 75% of heterozygosity in less than 50 generations. Our findings suggest that anthropogenic modifications on native forests, increasingly ongoing in Northeastern Argentina, Southern Paraguay and Southeastern Brazil, might prevent the dispersal of howlers, leading to population isolation. To ensure long-term viability and maintain genetic connectivity of *A*. *caraya* remnant populations, we recommend preserving and restoring habitat continuity. To conserve the species genetic pool, as well, the four genetic clusters identified here should be considered separate Management Units and given high conservation priority. In light of our findings and considering complementary non-genetic information, we suggest upgrading the international conservation status of *A*. *caraya* to “Vulnerable”.

## Introduction

Howler monkeys (Primates: Atelidae) are amongst the largest New World monkeys, inhabitants of several Neotropical ecoregions, from central Mexico to northeastern Argentina [1]. Nowadays, these Primates are being increasingly affected by anthropogenic activities, such as deforestation for agriculture and cattle ranching, and flooding of large areas for dam building which derive in loss, modification, reduction or isolation of native forest habitats [2–5]. Such changes trigger secondary processes in primate populations including dispersal restrictions, resource depletion, and pathogen exposure [6–9], which can reduce genetic diversity and effective population size, decreasing the adaptive potential of populations, increasing local extinction risks, and affecting the long-term survival of species [10]. If distance between habitat patches or modification of natural landscape structure prevents individual dispersal, gene flow between populations can be prevented, compromising the adaptation capacity and survival of the species in the long-term [10]. Population genetics can therefore provide insights into how anthropogenic changes affect primate populations, as well as into the historical and contemporary processes that shape the population structure [10], including the influence of landscape features on gene flow [8]. Describing the patterns of distribution of genetic diversity can help define population units important for effective management and conservation [10]. Moreover, population genetic parameters can be used in a holistic framework to support recommendations regarding official international conservation rankings [11]. To date, while some primate groups have been more explored regarding their genetic structure, others remain poorly studied [12]. Relative to other primates, Neotropical species represent an understudied group regarding population genetics [13]. New studies can help deepen our understanding on the factors that influence gene flow in these primates.

**Fig 1:**
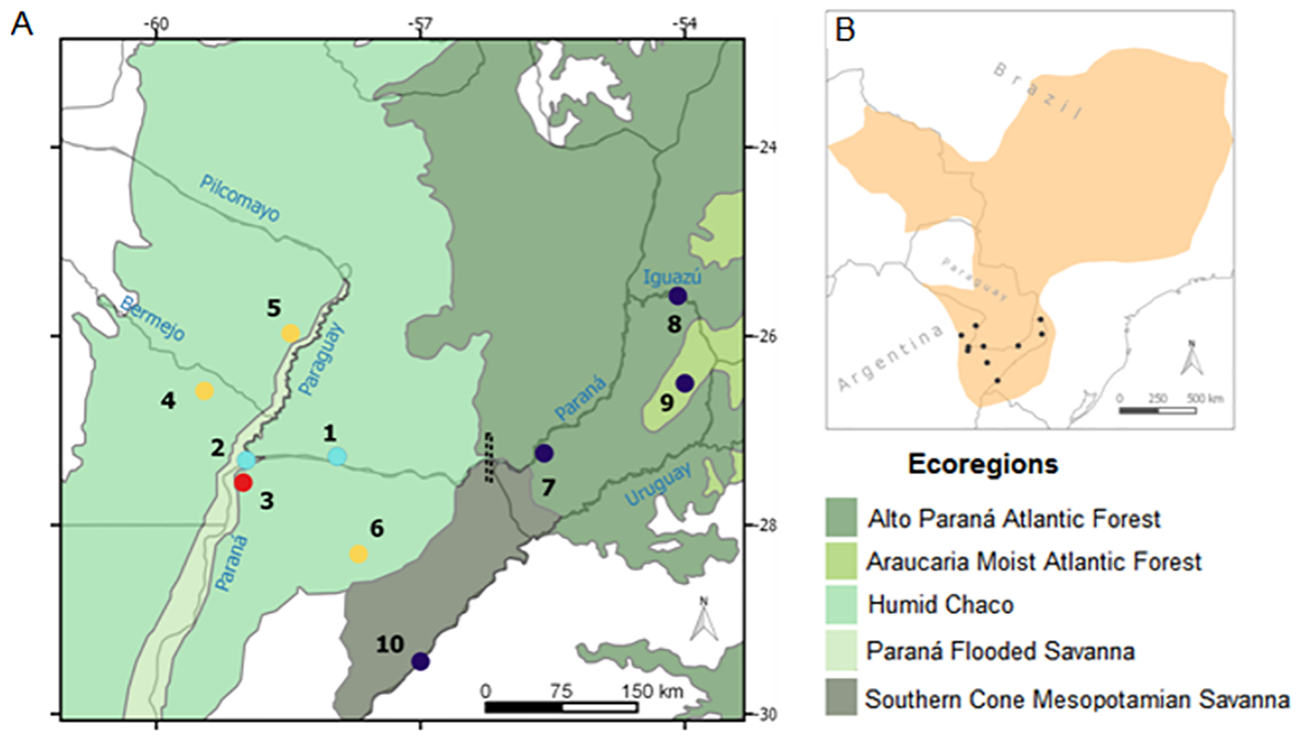
Map of sampling sites. Maps showing: (A) black-and-gold howler monkeys *Alouatta caraya* sampling locations in Northeastern Argentina and Southern Paraguay; populations (1 to 10) are represented by circles color-coded to mirror their allocation to the four main genetic clusters identified by Structure analyses. The different background colors indicate different ecoregions [31]. Main rivers are shown in blue font. The dotted black bar next to population 7 indicates the location of the Yacyretá Hydroelectric Dam; (B) Map showing the distribution range of BGHM modified from IUCN database with recent data from [17], with black dots showing location of sampling sites. Full names of sampling sites are given in Table 1.

Here, we focus on black-and-gold howler monkeys (*Alouatta caraya*; hereafter denoted as BGHM), arboreal primates which inhabit several ecoregions in South America (Fig 1). Some of these ecorregions, such as the Dry and Humid Chaco forests in Bolivia, Paraguay and Argentina [2, 3], and the Atlantic Forest in Brazil, Paraguay and Argentina [5], are subjected to major anthropogenic modifications, and are entirely fragmented, with native remnants mostly isolated. Population densities and social organization of BGHM differ remarkably along their distribution area [14]. Previous studies indicate that BGHM disperse through riparian forests, which act as biological corridors [15, 16], but habitat fragmentation severely limits their ability to disperse [7]. Demographic records show that both females and males leave their natal groups (social units); therefore, within groups, the adults are expected to be unrelated [17]. Although BGHM can survive in fragmented and impoverished habitats, including those that have undergone selective logging [18], indiscriminate deforestation and destruction of riparian forests could threaten their survival at the southernmost part of the species range [19, 20].

BGHM have major epidemiological importance because they are sensitive to the yellow fever virus and show high mortality when infected, therefore acting as early sentinels for virus detection [21]. BGHM abundance in the Atlantic Forest of Argentina drastically dropped after the 2008–2009 sylvatic yellow fever outbreak [22] and a recent study reported no evidence of the presence of BGHM in this area [15]. In southern Brazil, as well, the same outbreak decimated many BGHM populations [23], and a recent outbreak in February 2017 caused thousands of monkeys’ deaths [24, 25]. Currently, BGHM are globally classified as “Least Concern” [26]. However, they are being increasingly affected by loss and modification of their native habitat, hunting and trafficking for pet trade, and thus, considered “Near Threatened” in Brazil [27] and Bolivia [28]. Moreover, in the southern limit of the specie’ range (Fig 1), BGHM are classified even under higher risk categories, such as “Vulnerable” in Argentina [1], and “Endangered” in southern Brazilian states [27], highlighting the effects of increasing deforestation and the vulnerability of the monkeys to the yellow fever epidemics. Under such degree of pathogen exposure and habitat degradation, the long-term persistence of BGHM populations is of high concern. In this sense, assessing the levels of genetic diversity and gene flow in small and geographically distant remaining wild BGHM populations is fundamental to support their conservation as well as to clarify the international ranking of the species.

In this study, we used nuclear and mitochondrial markers to investigate ten BGHM populations inhabiting different ecoregions of northeastern Argentina and southern Paraguay (Fig 1), and subject to different degrees of habitat loss, modification and degradation. Our specific objectives were to: (1) describe past- and present-day levels of genetic diversity; (2) assess historical and contemporary patterns of genetic structure; (3) investigate possible drivers of genetic structure, specifically, geographic distance, differences between ecoregions, or variable levels of habitat loss and modification; and (4) provide conservation guidelines and key information for management or reintroduction projects involving these Neotropical primates, by, for example, identifying Management Units (MUs) [29]. Given that historical dispersal routes of BGHM have supposedly gone through unflooded and seasonally flooded riparian forests [16], populations connected by rivers in the past are expected to share mitochondrial similarities. Moreover, given that BGHM disperse through continuous forests, effective dispersal will be likely affected by anthropogenic modifications. Arboreal primates and non-primates are expected to depend on forest continuity to disperse. Previous studies in BGHM occupying a fragmented habitat interrupted by grassland extensions suggest a reduction in the dispersal rate between groups residing at distances greater than 1000 m [7]. Therefore, landscape modification, such as crop monocultures or grasslands used for cattle ranching, may isolate populations because dispersing individuals from their natal groups would have to descend to the ground, being highly susceptible to predation or other sources of mortality before reaching another fragment [7, 30]. Thus, populations of BGHM not connected by continuous forests are expected to show restricted gene flow and to be genetically different from one another.

## Material and methods

### Ethics statement

This study was carried out in strict accordance with Argentinean laws for research on non-human primates, and following the recommendations of ‘Principles for the Ethical Treatment of Primates’ of the American Society of Primatologists (available at: https://www.asp.org/society/resolutions/EthicalTreatmentOfNonHumanPrimates.cfm). We received specific approval to conduct this study by the Consejo Nacional de Investigaciones Científicas y Técnicas (CONICET) from Argentina (no. 11420110100322CO). Additional specific sampling permits were obtained from Chaco and Corrientes Provinces, Argentina (Permit Number: 01071), and from Ministry of Ecology, Misiones Province, Argentina (Permit Number: permit no. 04/15); tissue samples were collected as part of an investigation conducted jointly by the Global Health Program, Wildlife Conservation Society and the Ministry of Ecology, Misiones Province, Argentina (Permit Number: 304/09). All methods used for tissue sampling complied with the guidelines recommended by the Protocol for Primate Sample Methods (available from: http://www.vetmed.ucdavis.edu/ohi/local_resources/pdfs/PREDICT_Protocol_Primate_Sampling_29Feb12.pdf). Fecal collection was conducted without capturing the animals and therefore does not cause any harm to the studied species. The specific coordinates for each sampling location are: Pop1: 27,275°S 57,684°W; Pop2: 27,314°S, 58,646°W; Pop3: 27,550°S, 58,679°W; Pop4: 26,791°S, 59,631°W; Pop5: 25,970°S, 58,177°W; Pop6: 28,307°S, 57,457°W; Pop7: 27,467°S, 55,827°W; Pop8: 25,574°S, 54,075°W; Pop9: 26,500°S, 53,833°W; Pop10: 29,445°S, 56,800°W.

### Biological sampling and DNA extraction

We sampled 163 BGHM from ten populations inhabiting five ecoregions [31] (Fig 1), in the southernmost edge of the species’ geographic range in northeastern Argentina and southern Paraguay, subjected to different types and degrees of environmental modification. To minimize the sampling of relatives, as BGHM generally disperse during juvenile stages [30], two fecal samples were collected from each adult. In groups 4, 5, 6, 8, 9 and 10, feces were collected immediately after defecation. In group 7, tissue samples were collected from individuals found dead and kept refrigerated at 4°C until necropsy. All samples were preserved at 24°C in 50 ml screw-top tubes containing solid NaCl [32] until DNA extraction (three months to one year later). DNA was extracted from feces using the QIAamp DNA Stool Mini Kit, (QIAGEN, Valencia, USA), following the manufacturer’s protocol with slight modifications, and from tissue samples using standard SDS/Proteinase K digestion followed by phenol–chloroform organic extraction [33]. Appropriate precautions were taken to avoid sample contamination: every step of the experiment was performed in designated laboratory spaces, under laminar flow (with negative pressure) conditions, and using aerosol-resistant filter tips.

### Microsatellites amplification

Genotypes from populations 1, 2 and 3 were obtained in previous studies [7, 32]. The remainder samples were amplified at ten autosomal STR markers, previously used in studies in BHGM [34] (AC14, AC17, AC 45, D8S165, D17S804, LL1118, LL157, Tgms1, Tgms2 and AB7), following a two-step multiplex PCR method [35] with minor modifications, in a 25-μL final volume, with 50 ng of DNA (including negative controls with no template DNA),1xGoTaqbuffer (Promega, USA), 1.75 mM MgCl_2_, 0.2 mM of each dNTP, 1 U GoTaq DNA polymerase (Promega, USA), 4 pmol of each forward primer with an M13 tail, 4 pmol of each reverse primer, and 0.4 mg of Bovine Serum Albumin (BSA, Promega, USA). The second step consisted of a 12.5-μL reaction with 5 μL of a 5:100 dilutions of the first multiplex PCR product as template, 0.875 mM MgCl_2_, 4 pmol of a forward primer with an M13 tail, 4 pmol reverse primer, and 4 pmol 5´FAM or HEX-labeled M13 primer [34]. Cycling parameters were: initial denaturation (95°C for 4 min), followed by 35 cycles of denaturation (94°C for 45 s), annealing (90 s at 58–60°C), and extension (72°C for 60 s), and a final extension (5 min at 72°C) [34]. Products from the second amplification step of different markers, labeled with different fluorochromes, were combined and separated by electrophoresis on an ABI PRISM 310 Genetic Analyzer. Alleles were manually scored by visual inspection of electropherograms after developing of the bin panel for each locus in GeneMapper ID-X v. 1.2 (Applied Biosystems), using HD400-ROX as internal size standard. To detect possible genotyping errors due to allelic, three independent amplification reactions were performed for each DNA extract (totaling six independent PCRs per marker, per individual). Each homozygous genotype was re-amplified and genotyped three additional times, from the two separate fecal samples per individual.

### Mitochondrial DNA amplification

A491-bp fragment of the left domain of the mitochondrial DNA Control Region (mtDNA, CR) was amplified using primers How RA-1 (5’-CTACCATCAACACCCAAAGC-3’) [16] and RC-BugioR (5’-CCAGGTTAAGAGGGTGATAGC-3’, this paper). Amplifications were performed at a final volume of 25 μL, containing 25 ng of single DNA extractions,1x GoTaq buffer (Promega, USA), 1.75 mm MgCl_2_, 0.2 mM of each dNTP, 1 U GoTaq DNA polymerase (Promega, USA), 4 pmol of each primer, and 0.4 mg BSA. Cycling parameters were: initial denaturation (5 min at 94°C), followed by 35–40 cycles of denaturation (1 min at 94°C), annealing (30 s at 50°C), extension (1 min at 72°C), and a final extension(3 min at 72°C). All products were sequenced bi-directionally in an Applied Biosystems 3500 Genetic Analyzer using BigDye^®^ Terminator v3.1 Cycle Sequencing Kit (Applied Biosystems, Foster City, CA, USA), and with the samereverse and forward primers used foramplifications. Quality of the sequences was eyed-checked inspecting the electropherograms in Sequencher 5.3 software (LifeCodes, USA).Sequences were edited and aligned using the Musclealgorithm [36] in MEGAv6.0 [37]. As in [38], the possible presence of nuclear mitochondrial insertions (NUMTS) was inspected by performing a BLASTn^®^ search in the National Center for Biotechnology Information (NCBI) website (https://blast.ncbi.nlm.nih.gov/Blast.cgi). For this search, the filters and mask options were clicked off, word size was set to a value of 28, match/mismatch scores were set to 1/-2 and gap creation/extension penalties were set to ´linear´.

### Statistical analysis of microsatellites

#### Genetic diversity, effective population size and demographic parameters

Genotypes were screened for null alleles, stuttering, or scoring errors using Micro-Checker v2.2.3 [39]. Conformation to Hardy-Weinberg equilibrium (HWE) and linkage disequilibrium (LD) was assessed performing exact tests in Genepop v4.2[40] with default settings for Markov chain parameters.The number of alleles per locus, number of private alleles, observed heterozygosity, unbiased expected heterozygosity, inbreeding coefficient, and the probability that two matching genotypes taken at random come from siblings(*P*_*ID-SIBS*_)[41] were computed in GenAlEx v6.5 [42]. To account for differing sample sizes, we computed a rarefied measure of allelic diversity (Allelic Richness) in Fstat v2.9.3.2[43], based on a standard sample size of *n* = 5, the smallest sample with complete genotypes at all loci (Paraguay sample, see Table 1). Statistical differences between groups regarding diversity statistics were evaluated using Kruskal-Wallis rank-sum tests in R statistical environment [44], applying Bonferroni correction to adjust significance levels for multiple comparisons. The pairwise relatedness estimator, *R*, of [45] was computed to identify first-order relatives (i.e, *R* ≥ 0.375) [46] that could lead to biased inferences of the population structure. When necessary, we randomly removed one individual from each of the highly related pairs and further analyzed the population genetic structure using the trimmed datasets (see below). The effective population size (*Ne*) of each group was estimated using a single-sample linkage disequilibrium method with jackknifing, as implemented in LD Ne v3.1 [47], for a minimum allele frequency of 0.05 [48]. The premise of the LD method is that the magnitude of the correlation between allele frequencies is a function of the effective population size and reflects the past finite population history; also, as a function of the sample size (*n*), the correlation in allelic frequencies arises from sampling a limited number of individuals from the population for estimating gene frequencies and disequilibrium [49]. The LD method for estimating *Ne* is based on the expectation that small populations accumulate more disequilibrium over time [49]. The method is robust to population size reductions and can be corrected for possible biases when the sample size is lower than the real *Ne* [47].

**Table 1:** Sampling information and summary estimates of diversity at ten microsatellites for black-and-gold howler monkeys. Type of habitat regarding tree-cover, population codes, names and number of sampled social units (groups) are given; *n*: number of samples analyzed (amplified at a minimum of seven *loci*); *Na*: number of different alleles, *AR*: allelic richness, *PA*: number of private alleles, *Ho*: observed heterozygosity ± standard deviation, _*U*_*He*: unbiased expected heterozygosity ± standard deviation, *F*_*IS*_: inbreeding coefficient, with an asterisk indicating the significant value after Bonferroni correction (adjusted significance level: 0.0005), and *P*_*ID-Sibs*_: multilocus probability that two matching genotypes taken at random come from siblings.

*Habitat*	*Code*	*Pop*. *name*	*Groups*	*n*	*Na*	*AR*	*PA*	*Ho*	*uHe*	*F*_*IS*_	*P*_*ID-Sibs*_
Modified	Pop 1	Paraguay	2	5	2.8 ± 0.44	2.59	1	0.38 ± 0.08	0.42 ± 0.07	0.11	0.009
Continuous	Pop 2	Isla Brasilera	7	38	5.0 ± 0.91	2.80	6	0.50 ± 0.08	0.50 ± 0.08	-0.08	0.003
Modified	Pop 3	EBCo	11	42	4.5 ± 0.93	2.60	2	0.46 ± 0.08	0.44 ± 0.07	-0.06	0.006
Modified	Pop 4	PN Chaco	4	8	2.7 ± 0.26	2.30	1	0.39 ± 0.06	0.39 ± 0.05	0.01	0.013
Modified	Pop 5	Guaycolec	6	10	3.5 ± 0.04	2.62	1	0.46 ± 0.06	0.46 ± 0.06	-0.01	0.005
Modified	Pop 6	San Alonso	5	9	2.3 ± 0.39	1.96	0	0.41 ± 0.10	0.32 ± 0.08	-0.26	0.003
Continuous	Pop 7	Garupá	3	6	2.7 ± 0.39	2.38	1	0.46 ± 0.10	0.36 ± 0.07	-0.24	0.002
Continuous	Pop 8	Yacutinga	2	4	2.1 ± 0.34	2.10	1	0.45 ± 0.11	0.35 ± 0.07	-0.30	0.002
Continuous	Pop 9	Piñalito	4	8	3.2 ± 0.44	2.70	2	0.34 ± 0.05	0.50 ± 0.05	0.29*	0.003
Modified	Pop 10	Yapeyú	4	8	3.0 ± 0.39	2.44	2	0.35 ± 0.10	0.37 ± 0.08	0.05	0.002

#### Genetic structure

We first investigated genetic structure using microsatellites genotypic data by running both individual- and population-based analyses, at fine and regional spatial scales. For example, we evaluated spatial genetic autocorrelation at a fine scale, using GenAlExv6.5. This method is appropriate when there is no *a priori* way to predict the genetic structure. Under restricted dispersal, we would expect a pattern of positive autocorrelation, where individual-by-individual genetic distances should be more similar at shorter geographical distances [50]. Geographical distance classes were chosen to ensure that the intervals included an even number of pairwise comparisons, ranging from the minimum (0–27 km, the first distance class, including comparison of dyads within the same site) to the maximum distance between sampling sites (578 km). Statistical significance was assessed with 10,000 random permutations and one-tailed probability tests, and 95% confidence intervals around the autocorrelation coefficient, *r*, were calculated with 10,000 bootstraps. The distance class at which *r* is no longer significant can be interpreted as an approximation of the extent of detectable positive spatial genetic structure [51]. In addition, to assess contemporary gene flow, we ran non-spatial Bayesian clustering using models in Structure v.2.3.4 [52]. A series of 20 independent runs per *K* (ranging from 1 to 10) was conducted using the admixture model with correlated allele frequencies, sampling locations as prior (LOCPRIOR), and 1,000,000 MCMC iterations after a burn-in of 50,000 replicates. Given our uneven sampling sizes we applied the correction method proposed by [53] to account for a possible downward bias in the number of genetic clusters recovered by Structure. For this, we analyzed: 1) the full dataset excluding first-order relatives (*n* = 138 individuals, 10 sites), and three trimmed datasets prepared to achieve more even sampling schemes; 2) a dataset obtained by randomly removing individual genotypes, without replacement, from the two best sampled populations to reach a sample size of 15 individuals in each, plus all other samples (*n* = 88, 10 sites); 3) the original dataset, removing the populations with *n* ≤ 6 (*n* = 123, seven sites); and 4) the sub-sampled even-sized reduced dataset, plus removing the populations with *n* ≤ 6 (*n* = 73, seven sites). For each one of these sampling strategies, runs were performed with the above-mentioned settings. We then collected the outputs and computed the corrected estimators *MedMeaK*, *MaxMeaK*, *MedMedK*, and *MaxMedK*[53] which represent the number of different clusters to which at least one of the populations (e.g., individuals grouped by sampling location) belongs to. These indexes are robust to uneven sampling schemes, and perform equally well or better than other commonly used clustering methods [53].For each sampling strategy, we computed all four estimators with population membership coefficient thresholds varying from 0.50 to 0.80. Then, to determine the number of clusters in our sample, we looked for the *K* identified by *MaxMedK* and *MedMedK* for a 0.80 threshold, as these should be more conservative, less influenced by the presence of migrants, and less affected by an incorrect *a priori* grouping of some individuals into populations [53]. We used Pophelper v.1 [54] to visualize the results of Structure analyses for the most likely number of *K*, identified as detailed above.

### Landscape analyses

We analyzed the possible influence of landscape features on the genetic structure of BGHM by conducting analyses based on current theory in Circuitscape v 4.0 [55]. For this, we generated raster resistance maps representing the difficulty of BGHM to disperse through different habitats. We drew raster maps with landscape cover of 2009 using data from the European Space Agency portal (http://due.esrin.esa.int/page_globcover.php). The elements of the landscape were classified into three categories: 1) “tree cover” (including both native forests and pine plantations, as these have been shown to be used by BGHM to disperse [7], 2) rivers, and 3) crops/grassland/roads. Accordingly, we assigned resistance values representing the cost of movements for BGHM through these features: a low-resistance value of one for tree-covered pixels, an intermediate-resistance value of 25 for river pixels, and a high-resistance of 50 for crops/grassland/roads pixels. Additional analyses changing these arbitrary values did not change our results significantly. We transformed resistance maps into pairwise resistance distances between sampling sites, using an 8-neighbours correction scheme. Then, using the ‘vegan’ v2.4–3 package [56] in R, we conducted Mantel tests between genetic (*Dps* = 1 –proportion of shared alleles between populations) and resistance distances, as well as partial Mantel tests between *Dps* and resistance, controlling for Euclidean distances (in kilometers, computed from geographical coordinates), and between *Dps* and geographic distance, partialling out the effects of resistance distances [57]. The proportion of shared alleles by pairs of populations was calculated with PopGenReport v2.2.2 package [58] in R. If landscape features influence gene flow, we would expect the Mantel statistic between *Dps* and resistance and the partial Mantel statistic (controlling for the effect of geographic distance) to be significant, whereas the partial Mantel statistic between *Dps* and geographic distance (controlling for resistance distances), to be non-significant [57]. Population-based gene flow measures (i.e., pairwise *F*_*ST*_ values) were also computed between all pairs of populations in Fstat v2.9.3.2.

### Statistical analysis of mitochondrial DNA

Haplotype frequencies, nucleotide composition, the number of transitions and transversions, and the number of polymorphic sites were calculated in Arlequin v3.522 [59]. Haplotypic (*h*) and nucleotidic diversity (*ð*) were calculated in DnaSP v5.0 [60]. Standard tests of selective neutrality, *R*_2_[61], Tajima’s D [62], and Fu's *F*_*S*_ [63], and their 95% confidence intervals, were conducted in DnaSP v5 with 1,000 simulations and a neutral infinite-sites model assuming a large constant population size. A constant population size represents the null hypothesis under the neutral model (i.e., the standard coalescent) [64]. Under the constant size hypothesis, when sample sizes are small, as is the case for some of our samples, the statistical tests Fu's *Fs* and *R*_*2*_ have more power to reject the null hypothesis [64]. Selective neutrality was rejected if small *R*_2_ and negative Fu's *F*_*S*_ values were significant. A Median-Joining haplotype network [65] was built in PopArt program [66]. To inspect for historical demographic processes undergone by the populations, we carried out an analysis of ‘mismatch distribution’ [67] in Arlequinv3.522with 10,000 bootstraps. Populations at a demographic equilibrium or declining are expected to exhibit a multimodal distribution pattern of pairwise differences between haplotypes, whereas populations that have experienced a sudden demographic expansion are expected to display a unimodal distribution [68]. The smoothness of the mismatch distribution curve was measured using the raggedness (*Rg*) and the Sums of Squared Deviations (*SSD*) indexes [68]. The significance of the test was evaluated through 1,000 coalescent simulations, assuming a neutral infinite-sites model and a constant population size. The timing of the demographic processes was estimated by computing *Tau* [67], using the formula τ = 2*ut*, where *u* is the mutation rate of the assayed fragment. We used 0.15 mutations per site per million years, as in previous studies in howler monkeys [16]. To express the time since the expansion in years, we used a generation time of 5 years, which is the average age of first breeding of BGHM [69]. Finally, for mtDNA sequence data, genetic structure was examined in Arlequinv3.522by conducting an analysis of molecular variance (AMOVA) [70] comparing all sampling sites separately, as well as a hierarchical AMOVA grouping the populations within five ecoregions (1. Humid Chaco, HC: Pop 1, 3, 4, 5 and 6; 2. Paraná Flooded Savanna, PFS: Pop 2; 3. Alto Paraná Atlantic Forest, APF: Pop 7 and 8; 4. Araucaria Moist Atlantic Forest, AMAF: Pop 9; and 5. Southern Cone Mesopotamian Savanna, SCMS: Pop 10, see Fig 1 for further reference), and computing *θ*_*ST*_ between populations and population groups.

## Results

### Genetic diversity, effective population size and demographic parameters

For most samples (*n* = 98) all ten loci were amplified, whereas in 40 samples a minimum of seven *loci* were amplified. We found no evidence of linkage between any pair of loci (*P*< 0.05), nor evidence of significant deviations from Hardy-Weinberg Equilibrium. We found evidence of first-order relatives (*R* ≥ 0.375) within populations (Pop) 2 and 3, and removed one individual from each of those dyads for further diversity and structuring analyses (final complete dataset *n* = 138). BGHM populations exhibited moderate levels of microsatellite diversity (Table 1). The mean number of alleles, *N*_*A*_, was3.16 ± 0.18; the mean allelic richness. *A*_*R*_, was2.93 ± 0.81, the lowest value was detected in Pop 6, whereas the highest in Pop 2, the latter also showing the highest number of private alleles. Mean unbiased expected heterozygosity (*uHe*) was 0.443±0.025 overall populations; *uHe* of Pop 2 differed significantly from *uHe* of Pop 6, 7, and 10, and *uHe* of Pop 9 differed significantly from that of Pop 7 and 8 (Kruskal-Wallis tests, p< 0.05). A significant signal of inbreeding was found only in Pop 9 (Table 1). *Ne* estimates were 40 (95%CI: 21–112) for Pop 2, and *Ne* = 26 (95%CI: 15–52) for Pop 3. The remainder populations yielded *Ne* with infinite 95% CIs.

**Fig 2:**
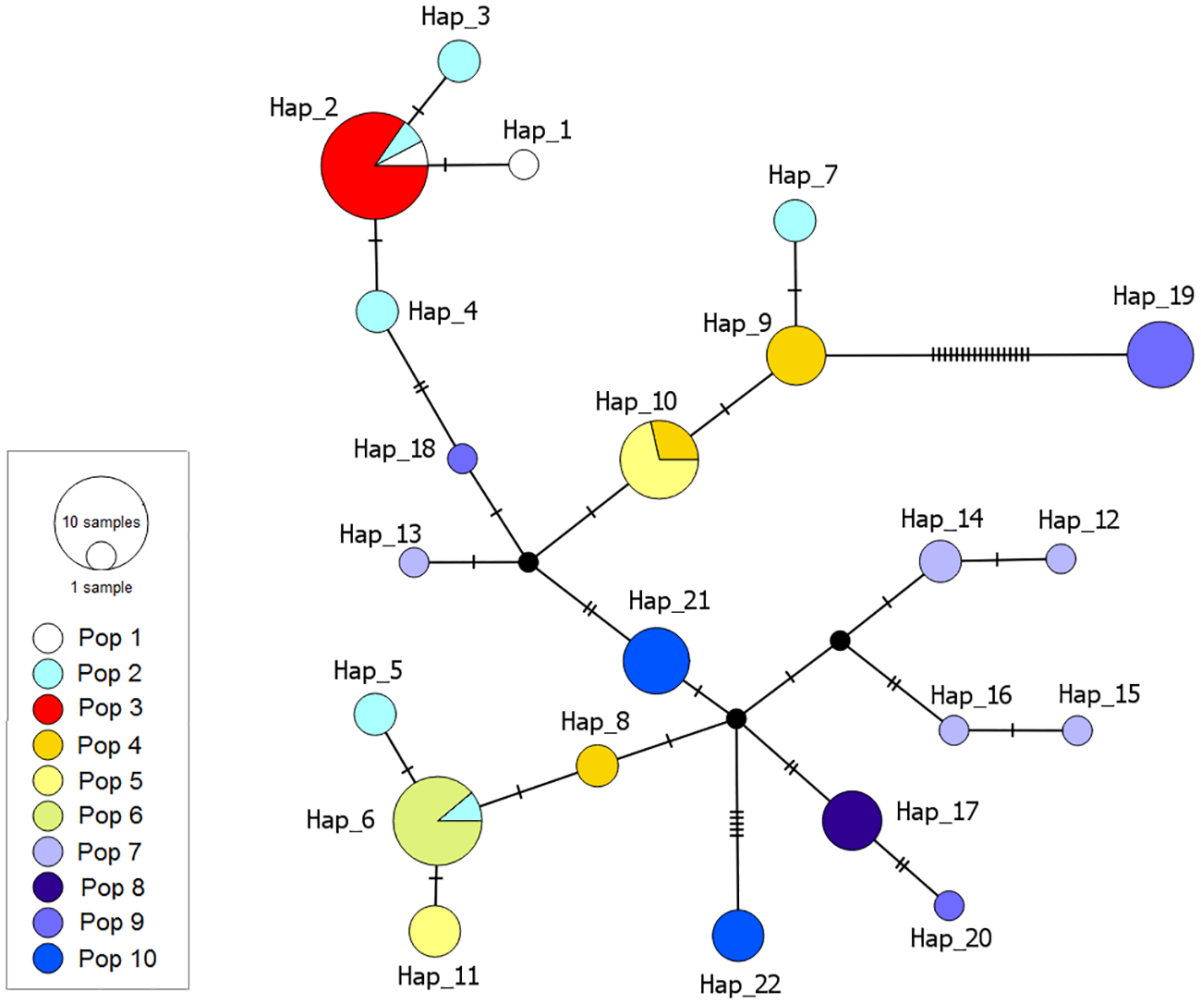
Haplotype network of black-and-gold howler monkeys. Median-Joining network of 22 haplotypes observed in black-and-gold howler monkeys from northeastern Argentina and southern Paraguay. Circle sizes are proportional to haplotypic frequencies; small black circles indicate median vectors; black lines indicate mutational steps. Full names of populations are given in Table 1, and colors approximate to those defining population clusters.

Thirty-six transitions defined 22 mtDNA CR haplotypes (*n* = 72), 15 of which were new to this study (Genbank accession numbers **MF095740, MF095741, MF095742, MF095743, MF095744, MF095745, MF095746, MF095747, MF095748, MF095749, MF095750, MF095751, MF095752, MF095753 and MF095754**), an overall haplotypic diversity of 0.930 *±* 0.11, and an overall nucleotidic diversity of 0.01*±* 0.007. Pop 2 was the most diverse in terms of number of haplotypes, but these were moderately divergent from each other (Fig 2, Table A in S1 File). Pop 3, 6, and 8 showed one haplotype each. Five individuals of Pop 9 showed one haplotype (H_19) separated by a minimum of 20 mutational steps from its ancestral haplotype (Fig 2). The BLAST search of the 491-bpmtDNA fragment of *Alouatta caraya* as a query sequence retrieved only mitochondrial sequences from Platyrrhini primates from Atelidae family (nine species) and Cebidae (three species) family. Identity of fragment sequences ranged from 77% to 99% with E-values ranging from 0.00 to 0.0057, respectively. Therefore, the presence of NUMTs in our fragment sequences can be considered negligible.

There was no correlation between mtDNA CR lineages and geographical distribution; some haplotypes were shared between geographically distant populations and different lineages occurred within a population (Fig 2). For the complete BGHM dataset, *D* (-0.071), *Fs* (-1.889), and *R*_*2*_(0.099) were non-significant, supporting the neutrality of the assayed fragment. The unimodal pattern of the mismatch distribution (data not shown) was consistent with a demographic expansion scenario estimated to have occurred 9,183 years ago (*Tau* = 7,053). At the population level, significant *SSD* and *Rg* were observed in Pop 5 and Pop 9, which could be interpreted as a demographic expansion (Table A in S1 File).

### Genetic structure

**Fig 3:**
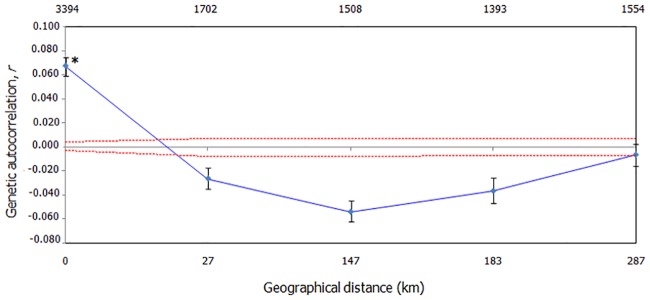
Spatial autocorrelation in black-and-gold howler monkeys. Spatial correlogram for howlers (*n* = 138) showing the genetic correlation coefficient (*r*) as a function of geographic distance across defined spatial distance classes. Dashed red lines represent upper (U) and lower (L) bounds of the null hypothesis of no spatial structure based on 10,000 random permutations. Error bars represent 95% confidence intervals about *r* based on 10,000 bootstraps. The asterisk denotes significantly positive *r* at α = 0.05. The number of pairwise comparisons within each distance class is shown above the plot.

**Fig 4:**
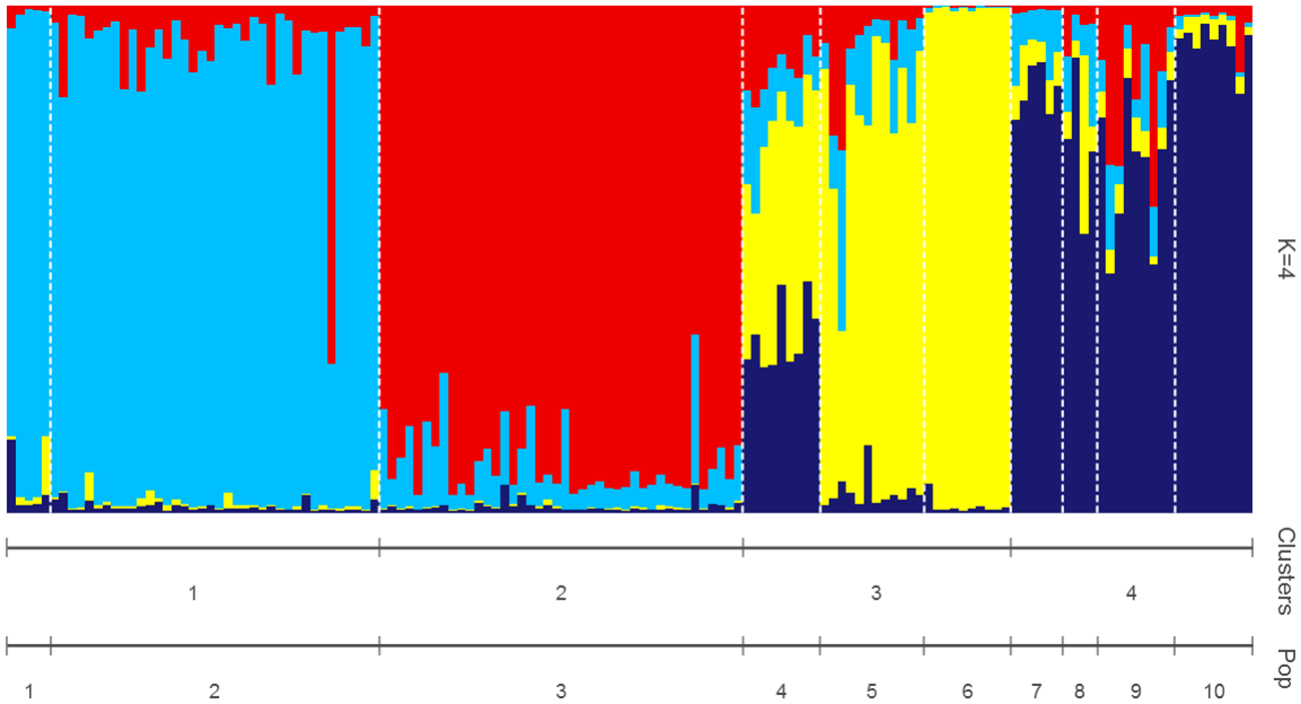
Genetic structure in black-and-gold howler monkeys. Membership bar-plots of black-and-gold howler monkeys (*n* = 138) sampled across ten sites in northeastern Argentina and southern Paraguay, resulting from Bayesian clustering analyses in Structure [52] based on genotypic data from 10 microsatellites. Individuals are represented by vertical lines (y-axis) broken into color-segments proportional to their membership coefficients to each cluster (*K* = 4), and were grouped into populations of sampling, separated with a white dashed line. Equally colored populations share genetic ancestry and are differentiated from the others. Full names of populations are given in Table 1.

**Fig 5:**
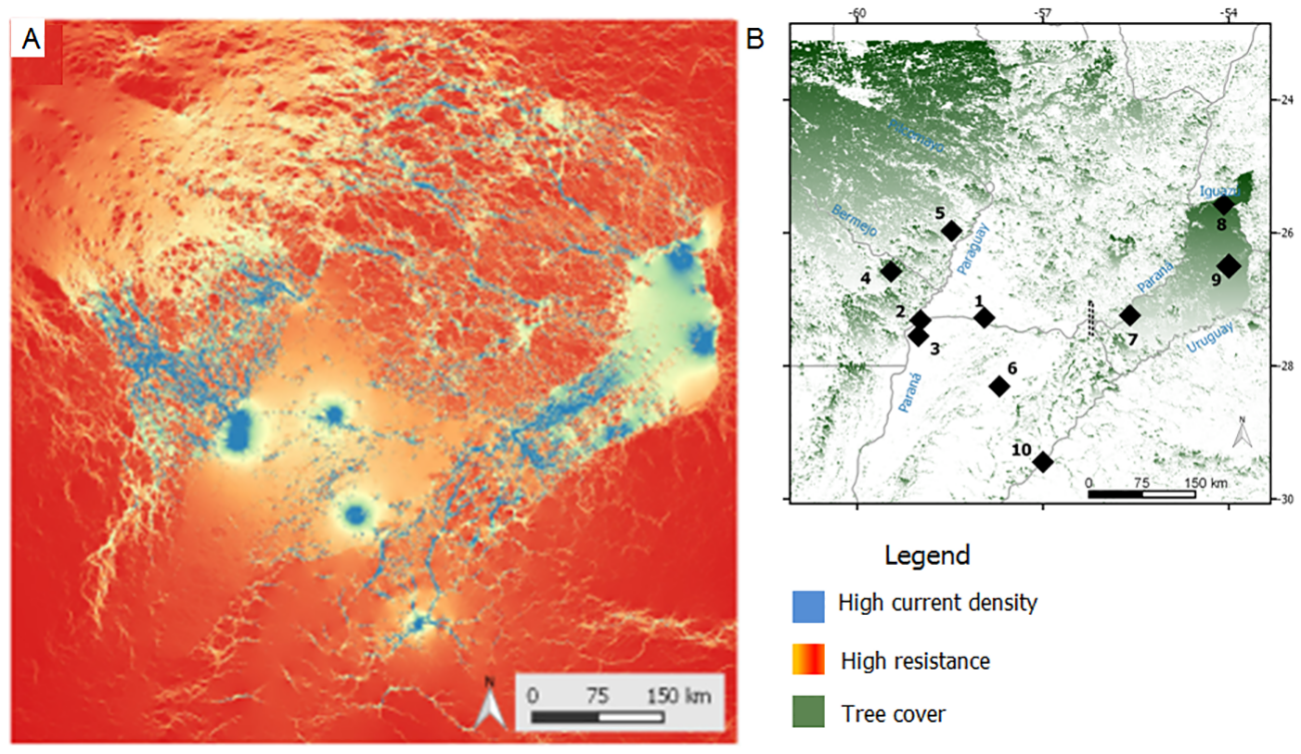
Maps of connectivity between populations of black-and-gold howler monkeys. Current maps of northeastern Argentina and southern Paraguay showing flow of current. Blue-colored areas represent highest current densities (higher connectivity) whereas light yellow-orange areas represent highest resistance (lowest current densities). Under the hypothesis of dispersal along tree-covered areas and riparian forests, areas in blue will therefore facilitate gene flow whereas areas in light yellow may restrict gene flow. Maps were generated in Circuitscape program, and re-colored in ArcGIS v 10.1 (http://www.esri.com/software/arcgis/arcgis-for-desktop); (B) Map of the studied region showing the 2009 tree cover surface used for Circuitscape analyses. For reference, the sampling sites are indicated with numbers (1–10).

The average inter-individual genetic distance was 7.0 ± 3.0. Results of spatial autocorrelation analyses revealed a significant positive spatial structure (*r* = 0.067, *P*< 0.01) only at the first distance class (0–27 km), indicating that BGHM were more closely related to other members of the same population than to BGHM from other populations (Fig 3). The results of the corrected Structure procedure identified four genetically differentiated population clusters as best explaining the nuclear genetic variation observed in BGHM. All datasets analyzed (i.e., the full corrected dataset as well as the sub-sampling strategies) resulted in the same number of clusters (Table B in S1 File). The plots of ancestry membership proportions showed that the four clusters comprise: i) Pop 1 and 2; ii) Pop 3; iii) Pop 4, 5, and 6; and iv) Pop 7, 8, 9, and 10 (Fig 4). In agreement, the maps produced in Circuitscape showed a path of low resistance comprising populations 7, 8, 9, and 10, and another comprising populations 2, 4, and 5, whereas populations 1, 3, and 6 remained more isolated from the others (Fig 5). The Mantel test between *Dps* and resistance distances was significant (*r* = 0.528, *P* = 0.006), while that between *Dps* and geographic distances was not (*r* = 0.267, *P* = 0.081). The partial Mantel test between *Dps* and resistance (partialling out geographic distance) was significant (*r* = 0.648, *P* = 0.001), while that between *Dps* and geographic distance (partialling out resistance) was not (*r* = 0.503, *P* = 0.061).

The global *F*_*ST*_ for microsatellites was 0.13 ± 0.34 and significantly different from zero (*p* = 0.001). Pairwise *F*_*ST*_ values between populations indicated substantial genetic differentiation, with 32 significant out of 45 comparisons (Table C in S1 File). Global *θ*_*ST*_ for mtDNA CR fragments was 0.18 ± 0.22 and significantly different from zero (*p* = 0.001). Pairwise *θ*_*ST*_ values showed that Pop 3 and Pop 6 differed significantly from the others, except for Pop 1 and 2; in turn, Pop 5 differed significantly from 6 and 10, and Pop 10 differed significantly from Pop 9 (Table C in S1 File). The hierarchical AMOVA for microsatellites grouping populations belonging to the same ecoregion showed that 6.20% of variation was significantly distributed among populations within ecoregions (*p*< 0.0001, *d*.*f*. = 5), and that 2.62% variation was significantly partitioned among ecoregions (*p*< 0.0001,*d*.*f*. = 4); likewise, pairwise *F*_*ST*_ values between ecoregions were significant, except for APF-AMAF and AMAF-SCMS comparisons. In agreement, results of the hierarchical AMOVA for mtDNA showed that 46.14% of variation was significantly distributed among populations within ecoregions (*p*< 0.0001, *d*.*f*. = 5) and that 11.56% of variation was significantly partitioned among ecoregions (*p*< 0.0001, *d*.*f*. = 4). Pairwise *θ*_*ST*_ values showed significant genetic differentiation (*p*< 0.0001) between AMAF and the other ecoregions, and between SCMS and all ecoregions except for PFS. The Mantel statistic for mtDNA genetic distance and geographic distance between populations was non-significant (null hypothesis: *r* ≤ 0; Z = -38.90, *r* = 0.0009, *p* = 0.476).

## Discussion

Loss or alteration of native forest and yellow fever outbreaks represent severe threats to black-and-gold howler monkeys in the southernmost limit of the species range. In light of this scenario, this study assessed their regional genetic diversity, gene flow and connectivity patterns, revealing that they conform four genetically differentiated clusters. Our results may contribute to the upgrade of BGHM conservation status, and provide guidelines for the future management of remnant populations.

### Genetic diversity and past demography of BGHM southernmost populations

In natural populations, genetic diversity provides the basis for the maintenance of evolutionary potential and adaptive capacity of individuals to face threats such as environmental change and disease [10]. The populations of black-and-gold howler monkeys studied here occupy sites in the southernmost part of the species’ range in northeastern Argentina and southern Paraguay (Fig 1) and showed mean microsatellites’ diversity (0.420 ± 0.082) similar to or lower than populations of congeneric species studied with some of the same markers employed here (*Alouatta pigra*: 0.430 [71] and 0.588 [72], *Alouatta belzebul*:0.640 [73], *Alouatta palliata*: 0.584 [74], suggesting that they are genetically impoverished, compared to other howlers. The population 2, located in the lower Paraná River, showed the highest genetic diversity estimates and shared haplotypes (Fig 2, Table A in S1 File), suggesting that it historically exchanged migrants with three other populations. Population 6, which inhabits the middle of the Iberá wetlands, showed the lowest genetic diversity estimates, suggesting that it could have been founded by a few individuals which faced dispersal limitations due to strong environmental restrictions. Population 9, located in the Argentinean Araucaria Moist Forest, showed five individuals with a unique, divergent haplotype (Fig 2), suggesting that it may be potentially reproductively isolated from the others. Alternatively, individuals with intermediate mitochondrial variants may have been extirpated by past yellow fever outbreaks in this region [22, 23]. BGHM from Pop 9 (sampled in 2006) showed a significant signal of inbreeding (Table 1), which could have been caused by genetic drift, suggesting that those howlers were already facing the erosion of genetic diversity, common to small endangered populations and, therefore, could have been immunologically depressed when the yellow fever virus attacked in 2007–2008. Our findings gain major importance in the context of recent virus outbreaks, since small populations of BGHM may exhibit increased susceptibility. In the next few decades, the high susceptibility of BGHM to yellow fever [24, 25] could act synergistically with other threats, putting these small and isolated populations at high extinction risk. It is worth noting that, in an epidemiological sense, the BGHM populations of humid Chaco are of utmost importance as genetic reservoirs of the species because yellow fever deaths have not been registered in this area during the last two episodes (2008/9-2017) [24, and Almeida MAB, pers. com.].

Past population expansion estimated with mitochondrial DNA data suggests that the populations located to the west of our sampling area expanded earlier than those located to the east (Fig 1, Table A in S1 File). Mismatch distribution results indicated that all populations expanded in South America during the post-glacial period (Last Glacial Maximum: 20,000–14,000 years ago, [75]). Moreover, the dating of the expansion event of populations 4 and 5 (Table A in S1 File), located in humid Chaco, is consistent with a recent comparative biogeographic study of neotropical primates, which suggests that most species currently inhabiting drier open habitats (such as the humid Chaco) have arrived there in the Pleistocene, from nearby rainforest habitats [76]. The lack of a strong evidence for demographic expansion in populations 7 and 8, located in the Alto Paraná Atlantic Forest, suggests demographic stability for this biome during the Pleistocene, in line with previous studies in other forest-dependent taxa inhabiting this region (e.g., birds, [77]). Conversely, Pop 9, which lies in the extreme west of the total distribution of the AMAF ecoregion (Fig 1), showed significant *SSD* and *Rg* indexes, which could be interpreted as a demographic expansion (Table A in S1 File), but more data are needed to explore this hypothesis.

### Genetic structure of BGHM southernmost populations

Black-and-gold howler monkeys are arboreal primates still present in some patchy and impoverished forests [19, 30]. The different analytical methods with varying assumptions employed in this study (Bayesian clustering, *F*_*ST*_, hierarchical AMOVA, and landscape analyses) were concordant in detecting significant present-day genetic structuring among the examined BGHM populations (Fig 4, Tables B and C in S1 File). Four distinct genetic clusters seem to best explain the nuclear diversity of BGHM inhabiting the southernmost part of the species range. The observed clustering pattern cannot be explained by an Isolation-by-Distance model (non-significant Mantel statistic), but, rather, seems to reflect the concurrent effects of multiple ecological, environmental and contemporary anthropogenic factors acting on the populations inhabiting different ecoregions. For example, populations 1 and 2, which share genetic ancestry and differentiate from others, are located nearby each other and connected by the riparian forest remnants of the Paraguay and Paraná Rivers (Fig 1).This result, as well as the mtDNA shared diversity and lack of historical differentiation among humid Chaco, Paraná Flooded Savanna and Alto Paraná Atlantic Forest (as indicated by *θ*_*ST*_) seem to support the hypothesis of dispersal via riparian forests, which act as biological corridors enabling the movement of BGHM [16]. Past immigration of BGHM through the riparian forests of the Paraná and Paraguay Rivers could have also contributed to the high genetic diversity observed in Pop 2, compared to other BGHM populations (Table 1) However, more recently, the movements of howlers through these dispersal routes may have been prevented by recent deforestation. In northeastern Argentina and southern Paraguay, the ecosystems were flooded as a consequence of the building of the Yacyretá Hydroelectric Dam in the 1970s [3,4], interrupting the terrestrial and riparian forest corridors that BGHM may have used in the past. In addition, we found that Pop 3, which lies in a remnant protected forest (Estación Biológica de Corrientes), surrounded by grassland, crops, and urbanized areas, clustered separately from all other populations. This significant genetic differentiation could have been caused a long history of forest exploitation and disturbance in this area, promoted by non-native human settlements documented since early 17^th^ century [18, 78]. Populations 4, 5 and 6, which inhabit patches of humid Chaco forest of different size and under variable degrees of protection, comprised another genetically differentiated cluster. Finally, populations 7, 8 and 9, which all lie in Misiones province in Argentina, upstream the Yacyretá Dam on the Paraná River, clustered together with Pop 10, located in Corrientes province, more distant but connected to the others via the Uruguay River riparian forest (Fig 1). Therefore, clusters 1, 2 and 3 involve populations placed upstream the Yacyretá Hydroelectric Dam on the Paraná River, while cluster 4 includes populations located downstream this dam. Misiones populations seem to maintain gene flow, but remain genetically isolated from more distant western populations that are immersed in more disturbed and isolated forest patches. This pattern of connectivity between nearby Misiones BGHM populations could be promoted via dispersal through a relatively well-preserved forest, as consequence of protection policies implemented in this province, such as the “Misiones Green Corridor” [79].

Landscape resistance seems to play a significant role in influencing the patterns of genetic structure observed in BGHM populations, as indicated by Circuistscape analyses (Fig 5), significant partial Mantel statistic between genetic distance and resistance, and non-significant partial Mantel statistic between genetic and geographic distance. The flow of the current showed in the maps (Fig 5) connects populations in a manner that corresponds to the four genetic clusters identified by microsatellites-based Bayesian approaches (Fig 4). Overall, our results suggest that crop/grassland fields could exert resistance to dispersal, and, consequently, to gene flow in BGHM. Populations connected by continuous forest or by relatively well-preserved riparian forests seem to share more similarities at nuclear loci than with populations immersed in more disturbed forests or with populations isolated by anthropogenic modifications, such as deforestation or building of dams. Therefore, our findings suggest that different levels of forest fragmentation that affect the studied populations may have exerted an important impact on the dispersal of the howlers, indicating that connectivity of the monkeys’ habitats is highly relevant for maintaining genetic connectivity across the landscape. These results highlight the importance of preserving continuous native forests, including riparian vegetation, for BGHM dispersal. In line with previous studies in other mammals inhabiting this region, our findings seem to indicate that anthropogenic modifications on native forests and depletion of continuous riparian forests, increasingly ongoing in northeastern Argentina, southern Paraguay and southeastern Brazil, prevent the dispersal of native fauna, and may lead to population isolation [80].

### Implications for the conservation and management of BGHM

Based on significant differences in allele frequency distributions and significant divergence in mitochondrial and nuclear loci, BGHM populations inhabiting northeastern Argentina and southern Paraguay comprise four different Management Units. Hence, we recommend that, to preserve the BGHM gene pool in the species’ southernmost range, these four main differentiated population clusters must be given high conservation priority. The pattern of significant genetic differentiation and restricted gene flow between BGHM populations revealed in the present study might result from increasing levels of forest loss and other anthropogenic modifications, such as the flooding of large habitat areas, derived from dam building, which severely limits the howlers’ ability to disperse and cross intermediate habitat regions [7]. In order to maintain or restore the natural gene flow between BGHM populations in the studied regions, the continuous forest patches, as well as the remaining riparian forests, should be protected and preserved. Further genetic studies spanning the entire geographical range of BGHM should help expand our knowledge on the patterns of gene flow and complement these conservation guidelines.

Based on the estimates of the effective population size obtained for the two biggest populations (*Ne* = 26 and 40), applying the equation for heterozygosity loss (Eqn. 4 in [11]), we can anticipate that the studied BGHM populations will lose heterozygosity below the 25% quantile of the current values in less than 50 generations. We also found that the populations at the southernmost limit of the species range have a reduced effective size and may be genetically depleted to face threatening events such as yellow fever outbreaks, which could rapidly affect all individuals in most of the species’ distribution area. Therefore, following the recently proposed genetic IUCN criterion (see Fig 2a in [11]), the studied BGHM populations should be classified as “Endangered”. We considered that the current IUCN global classification of BGHM as “Least Concern” [26] underestimates the treats to which each of the remaining BGHM populations are subjected; therefore, we propose a re-classification of the global status of the species to “Vulnerable”. This proposed global re-classification category is in line with country-level rankings of Argentina (“Vulnerable”) [81] and Brazil (“Near Threatened”) [27], that comply with the IUCN criteria “A4cd” which refers to a population reduction of 30% in 3 generations (4), where the reduction or its causes may not have ceased, may not be understood, or may not be reversible; mainly due to: (c) a decline in the area of occupancy, extent of occurrence, and/or quality of habitat, and (d) exploitation levels due to hunting or illegal traffic (pet trade). Brazil also adheres to the criterion (e) which refers to the effects of pollutants, introduced taxa, hybridization, competitors, pathogens, or parasites, referring to BGHM vulnerability to yellow fever epidemics. We recommend that, given the high susceptibility of black-and-gold howler monkeys to the yellow fever virus, this criterion should be also adopted by the international IUCN ranking. The current IUCN global status of *Alouatta caraya* seems to rely heavily on the species’ wide geographic distribution range (Fig 1), which includes large areas of unsuitable habitat and, therefore, does not adequately mirror the actual population density. Thereby, as it occurs with other taxa [82], the actual distribution range of BGHM is overestimated, while their level of risk is underestimated. Based on concurrent genetic and non-genetic evidence mentioned above, we recommend that the IUCN upgrades the global conservation status of *Alouatta caraya* to “Vulnerable”.

In sum, the present study contributes novel evidence supporting contemporary restricted gene flow between BGHM inhabiting the southernmost portion of the species’ geographic distribution range, and identifies four distinct Management Units for conservation. We also anticipate that most of the studied populations would loss heterozygosity in the mid-term and recommend that the IUCN global conservation status of BGHM is upgraded to “Vulnerable”. Lost habitat connectivity can play a significant role in preventing gene flow between isolated populations and, if not reverted, such a pattern may severely affect the survival capacity of BGHM. Our results have direct implications for the conservation of howlers and should be taken into account by policy makers when taking decisions, drafting management plans or designing reintroduction projects of these vulnerable Neotropical primates.

## Supporting information

S1 File **Table A. Summary estimates of mitochondrial diversity.** Genetic diversity estimates, results of neutrality tests and demographic parameters for black-and-gold howler monkeys sampled in ten populations from Northeastern Argentina and Southern Paraguay, based on 491-bp mtDNA Control Region fragment sequences. *H*: number of haplotypes (*n*: number of sequences). *PS*: number of polymorphic sites; *h*: haplotypic diversity± *standard deviation* (*SD*); *π*: nucleotidic diversity ± *standard deviation* (*SD*); Tajima’s (1989) *D*; Fu’s (1997) *Fs*; Ramos-Onsins and Rozas’s (2002) *R*_*2*_; *SSD*: Sum of Squared Deviations, *Rg*: Raggedness index; *Tau*: mode of the unimodal mismatch distribution; *TSE*: time since population expansion (in years before present). **Table B. Results of modified** **Structure** **procedure.** The most likely number of differentiated genetic groups (*K* = 4, column shaded in grey) found to better explain the variation observed in the genotypic dataset (ten microsatellite *loci*) of black-and-gold howler monkeys from Northeastern Argentina and Southern Paraguay. The Max-ofMedian (*MaxMedK*) and Median-of-Median (*MedMedK*) indexes (Puechmaille, 2016), taken at the 0.80 threshold of membership proportion, were used as conservative estimators of *K*. The different sampling schemes tested were: 1) the full dataset (*n* = 138, ten sites), 2) subsampling Pop 2 and 3 to obtain more even sample sizes (*n* = 88, ten sites), 3) original dataset but removing the least sampled populations *(n* = 123, seven sites), and 4) the dataset obtained by a combination of the two latter strategies (*n* = 73, seven sites) (see main text for further details). For each sampling scheme, 20 replicate STRUCTURE runs were performed from *K* = 2 to *K* = 10. **Table C. Genetic structure observed in populations of black-and-gold howler monkeys from Northeastern Argentina and Southern Paraguay**. Pairwise *F*_*ST*_ values for ten microsatellite loci (below diagonal) and pairwise *Φ*_*ST*_ values for 512-bp mtDNA Control Region fragment sequences (above diagonal). Significant values after Bonferroni correction are shown in bold (α = 0.0005).
(PDF)
