# Supplementary material for: Genetic structure in the southernmost populations of black-and-gold howler monkeys (Alouatta caraya) and its conservation implications
Source: PLoS One. 2017 Oct 2;12(10):e0185867. doi: 10.1371/journal.pone.0185867 (PMC5624639; doi:10.1371/journal.pone.0185867)
Supplement: S1 File — Table A. Summary estimates of mitochondrial diversity. Genetic diversity estimates, results of neutrality tests and demographic parameters for black-and-gold howler monkeys sampled in ten populations from Northeastern Argentina and Southern Paraguay, based on 491-bp mtDNA Control Region fragment sequences. H: number of haplotypes (n: number of sequences). PS: number of polymorphic sites; h: haplotypic diversity± standard deviation (SD); π: nucleotidic diversity ± standard deviation (SD); Tajima’s (1989) D; Fu’s (1997) Fs; Ramos-Onsins and Rozas’s (2002) R2; SSD: Sum of Squared Deviations, Rg: Raggedness index; Tau: mode of the unimodal mismatch distribution; TSE: time since population expansion (in years before present). Table B. Results of modified Structure procedure. The most likely number of differentiated genetic groups (K = 4, column shaded in grey) found to better explain the variation observed in the genotypic dataset (ten microsatellite loci) of black-and-gold howler monkeys from Northeastern Argentina and Southern Paraguay. The Max-ofMedian (MaxMedK) and Median-of-Median (MedMedK) indexes (Puechmaille, 2016), taken at the 0.80 threshold of membership proportion, were used as conservative estimators of K. The different sampling schemes tested were: 1) the full dataset (n = 138, ten sites), 2) subsampling Pop 2 and 3 to obtain more even sample sizes (n = 88, ten sites), 3) original dataset but removing the least sampled populations (n = 123, seven sites), and 4) the dataset obtained by a combination of the two latter strategies (n = 73, seven sites) (see main text for further details). For each sampling scheme, 20 replicate STRUCTURE runs were performed from K = 2 to K = 10. Table C. Genetic structure observed in populations of black-and-gold howler monkeys from Northeastern Argentina and Southern Paraguay. Pairwise FST values for ten microsatellite loci (below diagonal) and pairwise ΦST values for 512-bp mtDNA Control Region fragment sequences (above diagon [file pone.0185867.s001.pdf]

Supplementary material for online publication only

Genetic structure of southernmost populations of black-and-gold howler monkeys (*Alouatta caraya*) and its conservation implications.

Luciana I. Oklander, Carolina I. Miño, Gabriela Fernández, Mariela Caputo, Daniel Corach

Table A. **Summary estimates of mitochondrial diversity.**

Genetic diversity estimates, results of neutrality tests and demographic parameters for black-and-gold howler monkeys sampled in ten populations from Northeastern Argentina and Southern Paraguay, based on 491-bp mtDNA Control Region fragment sequences. *H*: number of haplotypes (*n*: number of sequences). *PS*: number of polymorphic sites; *h*: haplotypic diversity± *standard deviation (SD)*; π: nucleotidic diversity ± *standard deviation (SD)*; Tajima's (1989) *D*; Fu's (1997) *Fs*; Ramos-Onsins and Rozas's (2002) R_2; *SSD*: Sum of Squared Deviations, *Rg*: Raggedness index; *Tau*: mode of the unimodal mismatch distribution; *TSE*: time since population expansion (in years before present).

Statistic	**Pop 1**	**Pop 2**	**Pop 3**	**Pop 4**	**Pop 5**	**Pop 6**	**Pop 7**	**Pop 8**	**Pop 9**	**Pop 10**
H (n)	2 (2)	6 (10)	1 (11)	3 (8)	2 (8)	1 (8)	5 (6)	1(4)	3 (7)	2 (8)
PS	1	14	0	6	5	0	9	0	24	6
h ± SD	1	0.911 *± 0.035*	0	0.714 *± 0.123*	0.536 *± 0.123*	0	0.933 *± 0.122*	0	0.524 *± 0.209*	0.536 *± 0.123*
π *± SD*	0.001 *± 0.003*	0.012 *± 0.007*	0	0.005 *± 0.006*	0.005 *± 0.003*	0	0.008 *± 0.005*	0	0.019 *± 0.011*	0.006 *± 0.004*
D	-	1.199	-	0.806	1.764	-	-0.113	-	0.063	1.813
Fs	-	0.920	-	2.285	4.413	-	-0.807	-	5.712	5.011
R^2	0.500	0.223	-	0.226	0.268	-	0.203	-	0.176	0.268
SSD	0.000	0.039	0.000	0.111	**0.340**	0.000	0.028	0.000	**0.330**	0.000
Rg	0.000	0.060	0.000	0.204	0.790	0.000	0.093	0.000	0.685	0.789
Tau	0.000	9.797	0.000	7.072	6.211	0.000	5.328	0.000	0.000	0.000
TSE	-	12,756	-	9,208	8,087	-	6,937	-	-	-

Table B. **Results of modified STRUCTURE procedure.**
The most likely number of differentiated genetic groups ($K = 4$, column shaded in grey) found to better explain the variation observed in the genotypic dataset (ten microsatellite *loci*) of black-and-gold howler monkeys from Northeastern Argentina and Southern Paraguay. The Max-of-Median (*MaxMedK*) and Median-of-Median (*MedMedK)* indexes (Puechmaille, 2016), taken at the 0.80 threshold of membership proportion, were used as conservative estimators of *K*. The different sampling schemes tested were: 1) the full dataset ($n = 138$, ten sites), 2) subsampling Pop 2 and 3 to obtain more even sample sizes ($n = 88$, ten sites), 3) original dataset but removing the least sampled populations ($n = 123$, seven sites), and 4) the dataset obtained by a combination of the two latter strategies ($n = 73$, seven sites) (see main text for further details). For each sampling scheme, 20 replicate STRUCTURE runs were performed from $K = 2$ to $K = 10$.

Sampling strategy	Index	*K*=2	*K*=3	*K*=4	*K*=5	*K*=6
1 – Full dataset	*MaxMedK_0.8*	2	3	4	4	3
1 – Full dataset	*MedMedK_0.8*	2	3	4	4	2
2 – Even sample sizes	*MaxMedK_0.8*	2	3	4	5	3
2 – Even sample sizes	*MedMedK_0.8*	2	3	4	3	2
3 – Remove samples $n \leq 6$	*MaxMedK_0.8*	2	3	4	5	4
3 – Remove samples $n \leq 6$	*MedMedK_0.8*	2	3	4	4	3
4 – Even sample sizes + remove samples $n \leq 6$	*MaxMedK_0.8*	2	3	4	4	4
4 – Even sample sizes + remove samples $n \leq 6$	*MedMedK_0.8*	2	3	4	2.5	4

Table C. **Genetic structure observed in populations of black-and-gold howler monkeys from Northeastern Argentina and Southern Paraguay**.

Pairwise F_{ST} values for ten microsatellite *loci* (below diagonal) and pairwise Φ_{ST} values for 512-bp mtDNA Control Region fragment sequences (above diagonal). Significant values after Bonferroni correction are shown in bold ($\alpha = 0.0005$).

	Pop 1	Pop 2	Pop 3	Pop 4	Pop 5	Pop 6	Pop 7	Pop 8	Pop 9	Pop 10
Pop 1		-0.013	0.710	0.585	0.582	0.985	0.509	0.961	0.505	0.601
Pop 2	0.039		0.352	0.164	0.141	0.468	0.263	0.345	0.506	0.287
Pop 3	**0.142**	**0.077**		**0.815**	**0.814**	**1.000**	**0.802**	**1.000**	**0.760**	**0.818**
Pop 4	0.050	0.015	**0.075**		0.062	**0.741**	0.438	0.628	0.577	0.442
Pop 5	**0.105**	**0.053**	**0.114**	0.039		**0.675**	0.433	0.686	0.599	**0.449**
Pop 6	**0.261**	**0.133**	**0.177**	**0.183**	**0.107**		**0.662**	**1.000**	**0.709**	**0.643**
Pop 7	**0.075**	**0.071**	**0.153**	0.006	**0.097**	**0.241**		0.544	0.579	0.309
Pop 8	**0.119**	**0.080**	**0.177**	**0.092**	**0.081**	**0.244**	0.029		0.562	0.543
Pop 9	**0.133**	**0.046**	**0.109**	**0.095**	0.044	**0.200**	0.028	0.027		**0.622**
Pop 10	**0.131**	**0.110**	**0.171**	0.063	**0.087**	**0.262**	-0.014	0.070	0.061	
